# Hierarchy of Hybrid Materials—The Place of Inorganics-*in*-Organics in it, Their Composition and Applications

**DOI:** 10.3389/fchem.2019.00179

**Published:** 2019-04-04

**Authors:** Mariia S. Saveleva, Karaneh Eftekhari, Anatolii Abalymov, Timothy E. L. Douglas, Dmitry Volodkin, Bogdan V. Parakhonskiy, Andre G. Skirtach

**Affiliations:** ^1^Nano-BioTechnology Group, Department of Biotechnology, Faculty of Bioscience Engineering, Ghent University, Ghent, Belgium; ^2^Remote Controlled Theranostic Systems Lab, Educational Research Institute of Nanostructures and Biosystems, Saratov State University, Saratov, Russia; ^3^Engineering Department and Materials Science Institute (MSI), Lancaster University, Lancaster, United Kingdom; ^4^School of Science & Technology, Nottingham Trent University, Nottingham, United Kingdom

**Keywords:** inorganic, organic, nanoparticles, polymers, lipids, hybrid, hydrogels, cells

## Abstract

Hybrid materials, or hybrids incorporating both organic and inorganic constituents, are emerging as a very potent and promising class of materials due to the diverse, but complementary nature of the properties inherent of these different classes of materials. The complementarity leads to a perfect synergy of properties of desired material and eventually an end-product. The diversity of resultant properties and materials used in the construction of hybrids, leads to a very broad range of application areas generated by engaging very different research communities. We provide here a general classification of hybrid materials, wherein organics–*in*-inorganics (inorganic materials modified by organic moieties) are distinguished from inorganics–*in*–organics (organic materials or matrices modified by inorganic constituents). In the former area, the surface functionalization of colloids is distinguished as a stand-alone sub-area. The latter area—functionalization of organic materials by inorganic additives—is the focus of the current review. Inorganic constituents, often in the form of small particles or structures, are made of minerals, clays, semiconductors, metals, carbons, and ceramics. They are shown to be incorporated into organic matrices, which can be distinguished as two classes: chemical and biological. Chemical organic matrices include coatings, vehicles and capsules assembled into: hydrogels, layer-by-layer assembly, polymer brushes, block co-polymers and other assemblies. Biological organic matrices encompass bio-molecules (lipids, polysaccharides, proteins and enzymes, and nucleic acids) as well as higher level organisms: cells, bacteria, and microorganisms. In addition to providing details of the above classification and analysis of the composition of hybrids, we also highlight some antagonistic yin-&-yang properties of organic and inorganic materials, review applications and provide an outlook to emerging trends.

**Graphical Abstract:**
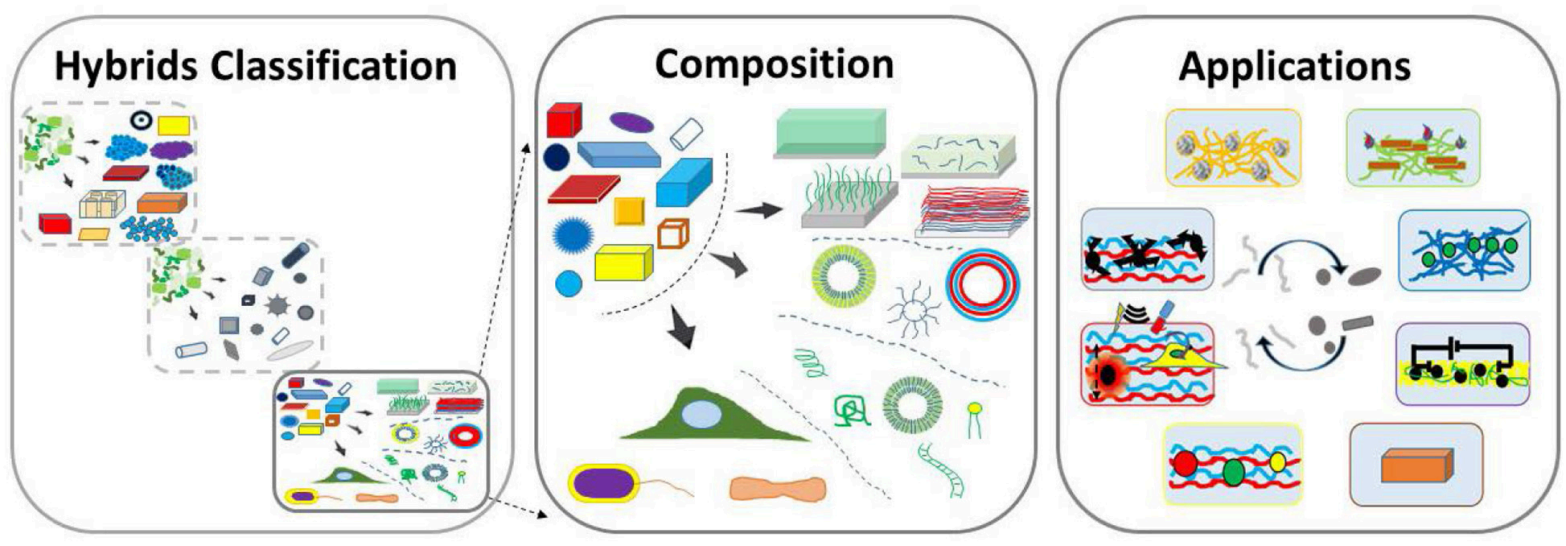
Hybrid Inorganics-in-Organics Materials.

## Introduction

The integration of both organic and inorganic materials is typically performed to improve properties or to obtain additional functionalities in resultant hybrid materials (Ruiz-Hitzky et al., 2008). A classic example here is the incorporation of hard and soft–two antagonist properties, which are very different in organic (soft) and inorganic (hard) materials—constituents into hybrids with a tunable stiffness of the resultant composite material. Interestingly, a combination of different materials has been present even from the time of ancient Maya, where hybrid pigments were formed (Sanchez et al., 2005). Different approaches to the classification of hybrid materials have been discussed. On the one hand, it can be based either on interactions (Sanchez and Soler-Illia, 2006), where those associated with van der Waals, hydrogen bonding, electrostatics are distinguished from those based on covalent and iono-covalent bonds, or, on the other hand, a distinction can be made based on their composition (Kickelbick, 2003). The area of hybrid materials is continuously and rapidly expanding, linking new research communities together with their own structures, specific subjects, and approaches. The resulting pool of the diversity of approaches is a potent catalyst to spur innovation, but developments in respective sub-areas may be, at least temporarily, overlooked by other research communities. Additionally, it takes a while to establish the same structural basis, common terms, interconnection and organization of broad and often still expanding research areas. This provides a general structural basis for the classification and organization of the overall hierarchy of hybrid materials.

In this regard, two distinct areas in the field of hybrid materials have been identified: modification of inorganic materials by organic molecules and, *vice versa*, modification of organic matrices by inorganic constituents. Overall, this can be structurally classified as follows:

1. Organic molecule-modified inorganic materials (organics-*in*-inorganics), which can be sub-divided:
  - inorganic structures modified by organic molecules;
  - colloidal particles stabilized by organic molecules.
2. Inorganic-modified organic materials (inorganics-*in*-organics).

**Figure 1:**
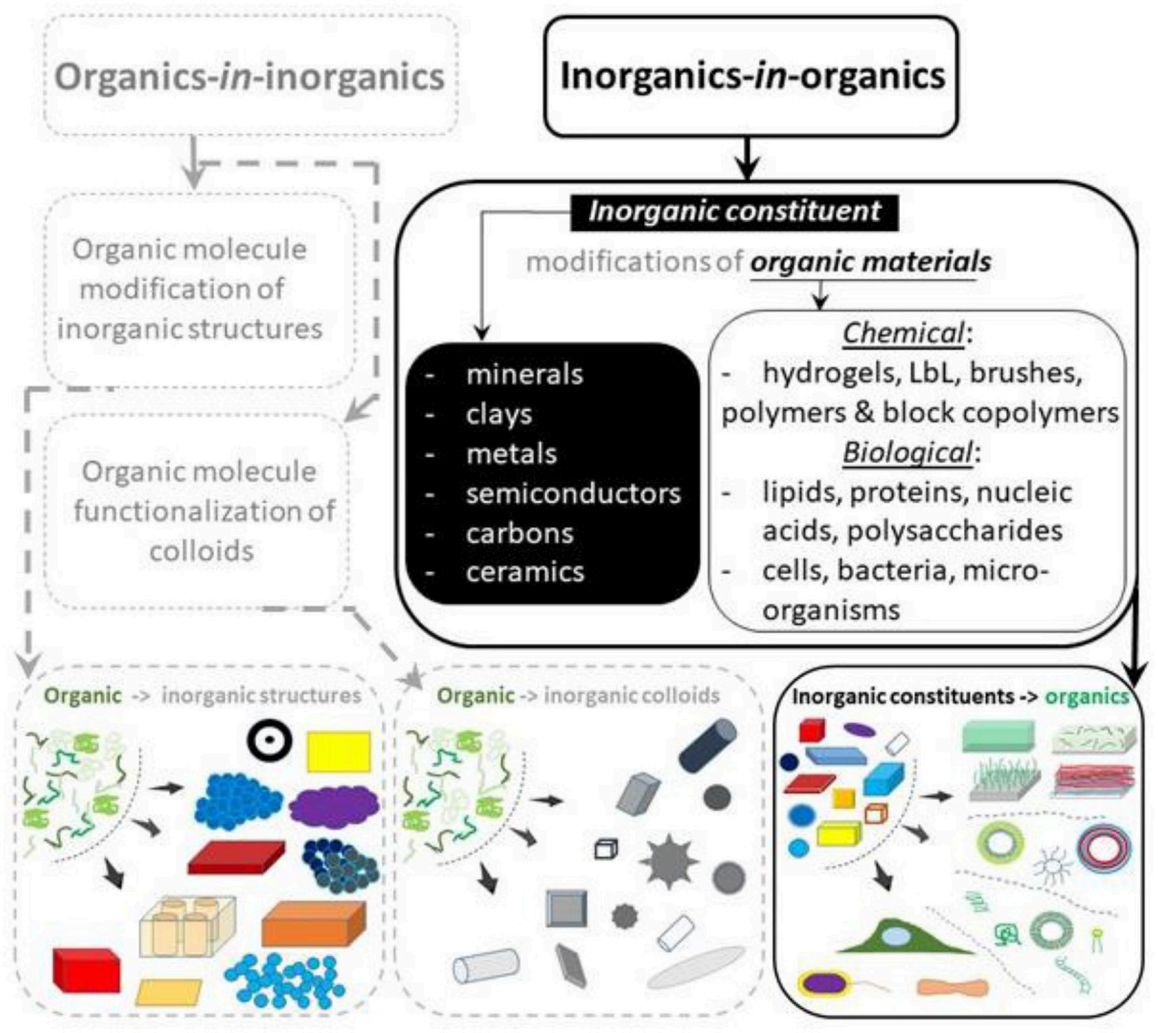
General classification of hybrid materials incorporating both organic and inorganic components. Functionalization of inorganic materials (the base material or matrix) by organic molecules, referred to as organics-in-organics, is shown on the left-hand side (shown in gray-dashed lines out outline the overall hierarchy of hybrids, but without being the focus of this research). Incorporation of inorganic constituents or components into organic materials (matrices) is referred to as inorganics-in-organics and is shown on the right-hand side (shown in solid dark lines, being the focus of this overview). The composition of inorganics-in-organics is outlined in a separate panel (right-hand side, in the middle). The bottom row depicts schematics of actual materials for each corresponding category of hybrids.

This structure is summarized in Graphical Abstract and is further reflected in Figure 1 with additional details. In the first (1a) application area, Figure 1 (left panel): modifications of inorganic content with organic molecules (organics), organics-*in*-inorganics, have been performed even with sol-gel hybrid nanocomposites, where the addition of organic and inorganic phases allowed combining complementary properties of these two classes of materials to produce those with a lower density and higher strength (Novak, 1993). Numerous applications, many of which have been commercialized to become household items, have emerged (Sanchez et al., 2005) and their number is continuing to grow. Generally speaking, another application sub-area (1b) can be viewed as a stand-alone sub-area, albeit that a clear distinction is not always made (Mir et al., 2018). First and foremost and in contrast to modifications in the first area (1a), where they (modifications) bring in additional properties, a functionalization of colloids by organic surfactants, with the exception of the electrostatic stabilization, is essential for their stability and it has become an inherent part of the ensuing research on small particles and clusters. It is therefore not surprising that colloidal science, which deals with inorganic nanoparticles, nanorods, nanotubes, nanostars, etc. and their stabilization, has become a distinct discipline. This area (1b), which can also be referred to as a surface modification or functionalization with ligands (Erathodiyil and Ying, 2011; Chanana and Liz-Marzan, 2012), is designated as a stand-alone sub-area (1b) in the classification chart, Figure 1 (bottom-row, in the middle).

In the last area (2), also depicted in Figure 1, inorganic modifiers such as colloidal (and Nano-) particles of minerals, clays metals, semiconductors, carbons, and ceramics are incorporated into organic materials of: (a) chemical (synthetic molecules, monomers, polymers, etc. as well as materials based on them: hydrogels, LbL, brushes, block copolymers both in the form of coatings and vehicles) or (b) biological origin (i.e., naturally occurring molecules, lipids, polysaccharides, proteins, nucleic acids including cells, bacteria, microorganisms). What further points to a distinct character of these areas is the fact that they are often developed by researchers with either inorganic chemistry or physical/organic chemistry backgrounds. It should be noted that organic-inorganic hybrids were described earlier (Chujo, 1996), but extensive systematic classification and organization needs to be updated.

This review focuses on the latter area (2): modification of organic matrices with inorganic components, as underlined by distinct solid black lines in Figure 1. We discuss their composition and highlight applications. The organic based materials, also referred to as matrices, are briefly introduced, highlighting the need for hybrids. Then, inorganic modifiers (inorganics) are briefly introduced identifying the range of properties they can enable. Hybrid materials for each class of organics are then described followed by a table-summary and conclusions with an outlook.

## Organic Matrices

Organic materials, also referred to as matrices, may play an important role in hybrid materials (Mastria et al., 2015), and they can be logically divided into chemical and biological materials.

**Figure 2:**
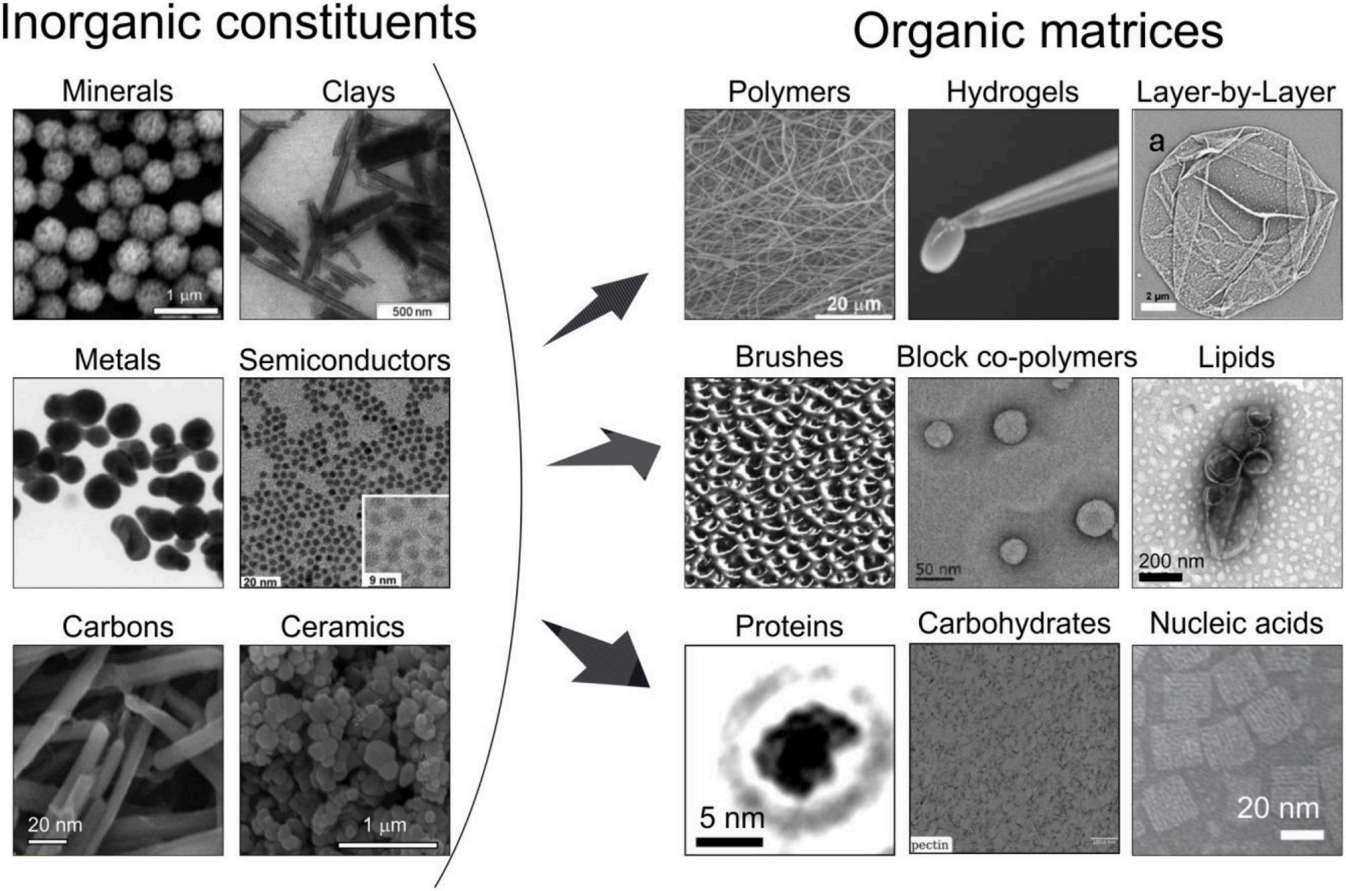
Classification of selected major classes of inorganic (left) and organic (right) components of hybrid materials as depicted by electron microscopy images. The inorganic constituents: minerals (SEM image of the calcium carbonate particles reproduced from Parakhonskiy et al., 2012 with permission Wiley-VCH), clays (TEM image of halloysites, reproduced from Fix et al., 2009 with permission Wiley-VCH), metals (TEM image of metal nanoparticles, reproduced from Simakin et al., 2019 with permission the ACS), semiconductors (TEM image of CdSe based nanocrystals, reproduced from Franzl et al., 2007), carbons (SEM image of carbon nanotubes, reproduced from Niazov-Elkan et al., 2018 with permission Wiley-VCH), ceramics (SEM images of TiO_2_, which is used in ceramics and reproduced from Weir et al., 2012 with permission of the ACS). The organic matrices are represented by the following chemical: polymers (SEM image of the polycaprolactone scaffold reproduced from Savelyeva et al., 2017 with permission Wiley-Blackwell), hydrogels (an optical photograph of the Image of an DNA hydrogel removed from atubeonapipette tip reproduced from Xu et al., 2017 with permission Wiley-VCH), LbL (SEM image of a polyelectrolyte capsule reproduced from Bedard et al., 2009c with permission the Royal Society of Chemistry), brushes (AFM image of the brush polymer film, reproduced from Lemieux et al., 2003 with permission of the ACS), block copolymers [TEM image micelles formed by amphiphilic diblock co-polymer poly(ethylene glycol)-block-polystyrene-PS310 reproduced from (Geng et al., 2016) with permission of Wiley-VCH]; and biological: lipids (TEM image of liposomes, reproduced from Ruozi et al., 2011 with permission of Dove Medical Press), proteins (TEM image of the BSA, reproduced from Longchamp et al., 2017 with permission of the Natl. Acad. Sci.), carbohydrates (TEM image of pectin, reproduced from Hernandez-Cerdan et al., 2018 with permission of the ACS), nucleic acids (TEM image of DNA brick Cuboid structure assembly, reproduced from Wei et al., 2014 with permission from Wiley-VCH) materials.

Organic chemical matrices are predominantly constructed from synthetic molecules, monomers, polymer-based materials structurally distinguished as coatings and vehicles, while compositionally assembled in the form of: hydrogels, layer-by-layer (LbL) assemblies, polymer brushes, and block copolymer based constructs. Some examples of inorganic constituents are presented in Figure 2 (left-hand panel) and of organic matrices in Figure 2 (right-hand panel).

## Organic Chemical Matrices

### Hydrogels

Hydrogels are polymer based materials, Figure 2 (right-hand panel) formed by cross-linked polymers leaving a substantial volume for water (Drury and Mooney, 2003). They can consist of networks of crosslinked hydrophilic polymers such as collagen, alginate, elastin, fibrin, etc. Cross-linking of hydrogels can be achieved by chemical methods: by aldehydes, addition and condensation reactions as well as physical methods: ionic interactions, crystallization. In addition, the following methods can be used for crosslinking: protein interactions, hydrogen bonds, reactions of amphiphilic block and graft copolymers (Hennink and Van Nostrum, 2002). Hydrogels are very versatile (Tokarev and Minko, 2010). The three-dimensional (3D) microenvironment of the hydrogel structures allows the supply of nutrients, gases, and wastes, as well as the delivery of active biomolecules, particularly important in tissue engineering and regenerative medicine (Stowers et al., 2015). Their properties can be precisely controlled in space and time (Place et al., 2009). The hydrogel stiffness influences cell behavior and can serve as a multidimensional cell culture platform to simulate tissue (Robitaille et al., 2013). The stiffness of hydrogels containing tissue/organ extracellular matrix supports cell morphology, while cell attachment, viability, and organization of the actin cytoskeleton can be controlled by adjusting the stiffness of hydrogels. It is desirable here to: control the stiffness, provide additional means for assembly, for example, biomineralization, and bring additional functionalities.

### Layer-By-Layer

Layer-by-Layer (LbL) assembly, Figure 2 (right-hand panel), has emerged as a simple and versatile method for coating biological and non-biological surfaces by alternatively depositing oppositely charged polyelectrolyte polymers (Decher, 1997). Its particular advantages are the flexibility to control the thickness, architecture, composition, and possibilities of incorporation of various materials (Lavalle et al., 2011) accompanied by various stimuli to control the properties (Delcea et al., 2011b). Research activities in the area of LbL include planar films (Von Klitzing, 2006; Selin et al., 2018) and capsules. In addition to nanometer-thin LbL films, so-called micrometer thick exponentially grown LbL films were developed (Lavalle et al., 2004), which can host an enormous amount of both small and high molecular weight substances due to a large thickness of multilayers, e.g., those made of biopolymers (Sustr et al., 2015, 2018; Velk et al., 2016; Vikulina et al., 2016; Prokopovic et al., 2017). The uptake of the molecules, with various natures, is driven by the molecule interaction with free (uncompensated) charges of the inter-polymer complexes in the multilayers. Cell adhesion to PEM films is mediated through electrostatic interactions and, more indirectly, via adsorbed serum proteins (Muller et al., 2010). The adsorption of enzymes was reported to be beneficial for cell growth (Liang et al., 2017). The amount of protein adsorption primarily depends on the final terminating layer (Wittmer et al., 2008), pH of the solution (Kreke et al., 2005), and the ionic strength (Ma et al., 2013b). In addition to the influence of electrostatic interactions, the cell adhesion increases with an increasing rigidity (Thompson et al., 2005). Soft and very hydrated multilayers can become cell-adhesive through the enhancement of mechanical properties via a coating with metal nanoparticles, or, cells can be localized into patterned multilayers made by microfluidics without any chemical or physical modification (Madaboosi et al., 2012a,b; Schmidt et al., 2012). Depositing semipermeable LbL layers onto colloidal particles has led to PEM capsules, which are freely suspended in a solution. Improving mechanical properties of PEM assemblies, adding sensory and remote release capabilities are desired functionalities in this area.

### Polymer Brushes

Polymer brushes or brushes, Figure 2 (right-hand panel) represent another type of coating. They are constructed using long-chain polymer molecules, in which one end is attached to a surface or interface. The density of attached polymers is typically high, forcing the chains to stretch away from the interface. Under these circumstances, the behavior of polymers, which can also be controlled by solvents, is different compared to that of flexible polymer chains in a solution. In some cases, diblock polymers can be used for the chain attachment between interfaces (Milner, 1991; Zhao and Brittain, 2000). It has been described that polymer brushes prepared from block polymers and synthesized by ionic polymerization can be absorbed onto flat substrates (Pyun and Matyjaszewski, 2001), while free-radical polymerization, which can be used to control the thickness, has also been used as a route to covalently bind polymer chains from surfaces with high grafting densities (Prucker and Ruhe, 1998). Atom transfer radical polymerization (ATRP) is known as a versatile technique for this purpose (Pyun et al., 2003), while thermal treatment of polymer brushes (Schroeder et al., 2018; Stetsyshyn et al., 2018) has been shown to affect the structure. Polymers can be tethered in a high density in an arrangement known as a bottle brush (Chremos and Douglas, 2018). Some examples of applications are the prevention of bacterial adherence, cell attachment, electrochemistry and the formation of colloidal crystals (Ayres, 2010), while additional functionalities are sought here.

### Block Copolymers

Block copolymers, Figure 2 (right-hand panel), represent a more general class of material assembled using polymers with at least two polymeric sub-units. Various polymerization routes, including atom transfer radical polymerization, addition-fragmentation chain transfer and ring-opening polymerization can be used to synthesize polymers with a tight polydispersity index and well-controlled molecular weight. Realizing that amphiphilic block copolymers can assemble in a similar fashion (as lipids form liposomes) has led to the research area of polymersomes (Discher and Eisenberg, 2002). What is particularly important is the fact that self-assembly of amphiphilic polymers (Zhao et al., 2013) can be made in such a way that the vesicles become even more stable than liposomes (Tanner et al., 2011). The area of polymersomes has seen a rapid growth and many new structures and assemblies, particularly relevant for biomedicine (Chécot et al., 2002; Palivan et al., 2016), have been designed (Cui et al., 2007; Christian et al., 2009; Van Oers et al., 2013). Furthermore, optimization of the loading efficiency has been done (Sanson et al., 2010) and various other structures, including micelles (Li et al., 2004) and micelles with different shapes (Wang et al., 2007) have been obtained. In a similar way to liposomes, membrane fusion has lso been demonstrated (Zhou and Yan, 2005). Various stimuli (Delcea et al., 2011b) can also be used in the area of polymersomes to control their properties (Che and Van Hest, 2016). Further development of responsive polymer vesicles is desired in this area.

## Organic Biological Matrices

Organic biological matrices are predominantly constructed from biologically relevant molecules including lipids, carbohydrates, proteins, nucleic acids (Cooper, 2000) as well as such higher level organisms as cells, bacteria, microorganisms. Some of the structures of organic biological materials are presented in Figure 2 (right-hand panel).

### Lipids

Lipid bilayers, also referred to as lipid membranes, are thin membranes comprised of two layers of lipid molecules (Nagle and Tristram-Nagle, 2000). Although lipid bilayer membranes undergo some changes of their state (Andersen and Koeppe, 2007), their permeability to molecules and ions is an essential functionality and has been the subject of intensive research. A particular relevance of lipid bilayers is associated with cells, because they form a continuous barrier surrounding cells providing the identity, communication with the environment, compartmentalization and protection (Hauser et al., 1972). It is due to these very important functions that the area of lipid bilayers is one of the most researched areas. The research area of liposomes (Pick et al., 2018)—small spherical vesicles comprised of lipids, which are used for the delivery of nutrients and nutrient supplements—is closely associated with lipids. Liposomes have always been important drug delivery carriers; they were reported to be multicarriers (Torchilin, 2006) capable of delivering doxorubicin, daunorubicin, and cisplatin. A desired functionality in the area of lipid membranes and liposomes is to control their permeability or to add functionalities.

### Proteins and Enzymes, Carbohydrates, Nucleic Acids

Proteins and enzymes, carbohydrates, nucleic acids and lipids are constituents of cells (Cooper, 2000). It can be noted that, on the one hand, these molecules constitute cells, while on the other hand, they can facilitate cell interaction with coatings through integrin-ligand interactions (e.g., collagen, fibrin, polypeptides) or other cell surface receptors (e.g., HA). Functionalization by these molecules is relevant for all coatings, including hydrogels (Devolder and Kong, 2012). Enzymatic functionalization of coatings is another important property (Sigolaeva et al., 2018), because enzymes are also excellent candidates to create tissue-like extracellular matrices (Caliari et al., 2016) and can even be used to encapsulate cells to create pre-seeded scaffolds (Hoffman, 2012). Drug delivery vehicles or coatings can directly be modified by inorganic constituents, mostly by nanostructures for sensing, enhancing mechanical properties (also in drug delivery), or promoting cell-surface interaction. Furthermore, a direct functionalization of molecules with inorganic nanostructures, nanoparticles and clusters can bring additional functionality in this area.

### Cells, Bacteria, Microorganisms

Cells, bacteria and microorganisms consist of organic molecules specified in the category organic biological matrices. But they are at a higher level of organization, making them stand in a separate category. In Figure 1, a subsection in the bottom-right panel points to the fact that this category stands apart in the overall organization. Both plant and mammalian cells (Cooper, 2000) as well as bacteria (Shapiro, 1998) are the building blocks of cell biology and microbiology. Single cells allow one to obtain essential information about fundamental processes in cell biology, while studying other multicellular organisms, i.e., bacteria, worms, insects, tissue, biofilms, and other microorganisms allows one to understand more sophisticated organisms.

## Inorganic Modifiers

Structurally, inorganic modifiers exist in many different shapes: particles of various shapes, coatings of different geometries as well as a variety of sizes ranging from clusters of atoms, nano-, and micro- particles to larger structures. The size is particularly essential, because they determine the overall properties due to specific effects associated with the behavior of electrons or induced charges.

Regarding compositionally, they can be divided into the following classes: minerals, clays, metals, semiconductors, carbons, ceramics as shown in Figure 2 (left-hand side). Minerals include a very broad range of materials, including rocks, stones, some oxides, and can either occur in nature or be synthesized (hydroxyapatites and carbonates, for biomineralization). Clays comprise of several groups: montmorillonite, kaolinite, Illite, chlorite. Hardness of clays is a property, which can be used in hybrid materials. Metals possess many attractive properties with their free electrons providing high electrical and thermal conductivity as well as enhanced absorption. At the nanoscale confinement comparable with the free electron path defines essential properties (Kelly et al., 2003). Semiconductors exhibit conductivity values between those of metals and insulators; the structure of their electron states makes them perfect candidates for sensing (Alivisatos, 2004). Carbon, as an element, is fairly abundant in the Earth's crust and universe. But what brings carbon to this list is its well-known forms: carbon nanotubes, graphene, carbon dots, whose absorption and conductivity make them attractive materials (Novoselov et al., 2004). Ceramics is another class of inorganic material with predominantly covalent or ionic bonds between atoms. Although many oxides constitute ceramics, its structure is generally more ordered than that of glass, which can be also considered here.

Various routes for assembling hybrid materials exist, including *in situ* synthesis (Adnan et al., 2018). But adsorption or interaction of already pre-made components is still frequently used in assembly by incorporating inorganic constituents in the form of nanoparticles, nanorods, particles, *etc*. and are comprised of the above-mentioned materials, Figure 2, into organic materials to obtain hybrids.

We note that the composition of the major classes provided in Figure 2 is not absolutely strict, but it provides a convenient way of classifying these major components of both classes of materials.

## Hybrid and Composite Materials

**Figure 3:**
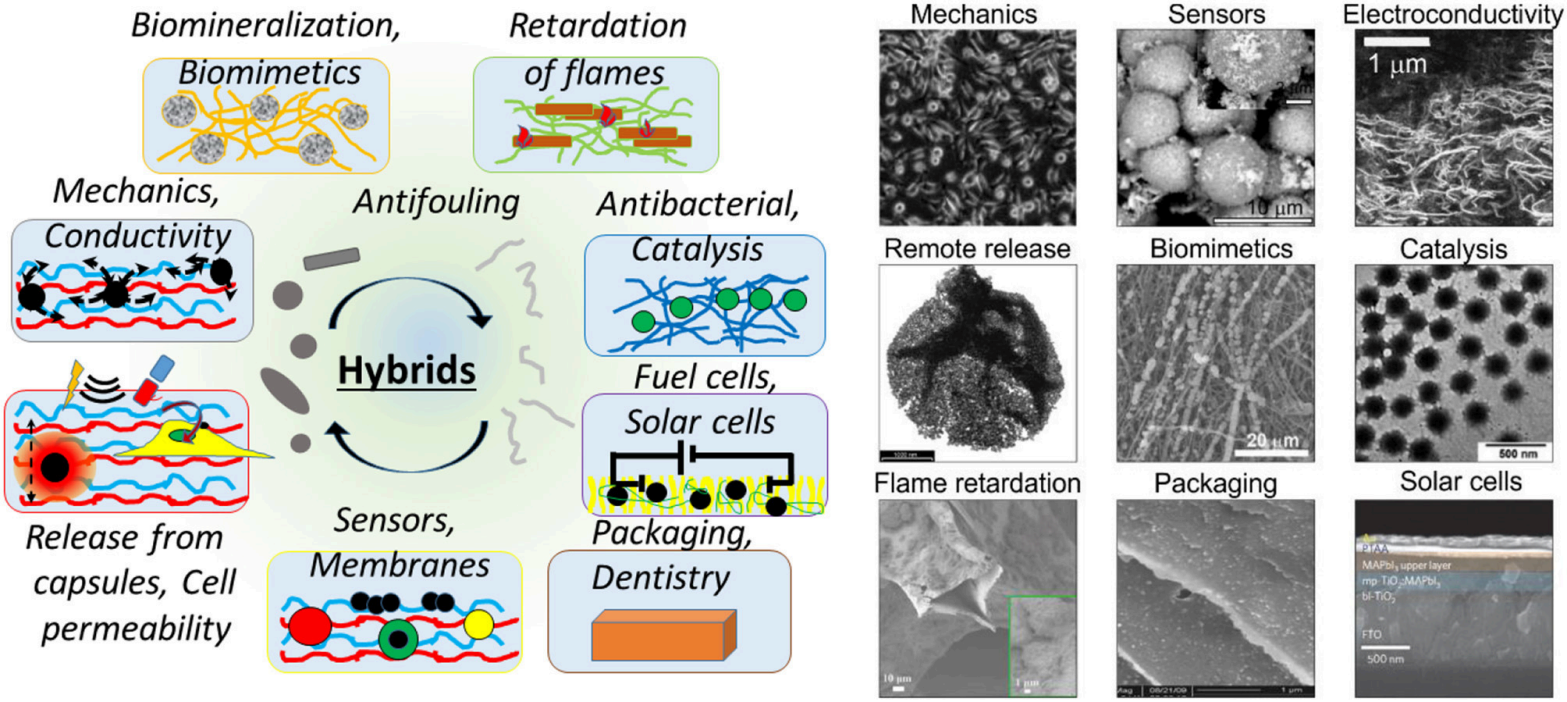
Various modifications of organic matrices by inorganic components classified according to their applications. The left-hand schematics shows a more general range of applications of hybrid materials, in which inorganic constituents are added to organic matrices, including: biomineralization, biomimetics, retartation of flames, antibacterial properties and catalysis, fuel and solar cells, packaging and applications in dentistry, sensors and membranes, release from drug delivery vehicles, cells or delivery into cells, enhancement of mechanical properties, electrical and thermal conductivity. The right-hand images illustrate selected objects assembled by incorporating inorganic constituents in organic materials for: enhancement of mechanical properties (Optical image of the cell adhesion behavior and the film surface morphology for different AuNP surface coverage, reproduced from Schmidt et al., 2012 with permission of the ACS), sensoric functions (SEM image of BSE on hydroxyapatite with silver nanoparticles as SERS platform, reproduced from Parakhonskiy et al., 2014 with permission of Elsevier Science BV), electroconductivity (SEM images of the surface of CNT/PS nanocomposites, reproduced from Grossiord et al., 2008 with permission of Wiley-VCH), remote release by an external action of a laser (TEM images of the shell of the polyelectrolyte capsule with Ag-nanoparticles, reproduced from Skirtach et al., 2004 with permission of the ACS); biomimetics (SEM image of the Polycaprolactone scaffolds mineralized with vaterite, reproduced from Savelyeva et al., 2017 with permission of Wiley-Blackwell), catalysis (TEM images of poly(N-vinylcaprolactam-co- acetoacetoxyethyl methacrylate-co-acrylic acid) P(VCL-AAEM-AAc) microgels reproduced from (Agrawal et al., 2013) with permission of the Royal Society of Chemistry), flame retardation (SEM images of polyurethane foam, with 3-bilayer halloysite nanotubes coatings, reproduced from Smith et al., 2018 with permission from the Wiley VCH); packaging (SEM images of zein-Kaolin nanocomposites containing 2.5% Kaolin, reproduced from Arora and Padua, 2010 with permission from Wiley-Blackwell); solar cells (cross-sectional SEM image of a complete perovskite device, reproduced from Jeon et al., 2014 with permission of Nature Publish. Group).

Some examples include the improvement or modification of mechanical properties and elasticity for cell adhesion, optical, catalytic and electrochemical properties, sensors, waterproofing, anticorrosion, insulation, etc. Figure 3 provides selected applications of inorganics-*in*-organics hybrid materials illustrating some images of the corresponding materials.

The diverse range of applications shown in Figure 3 is a result of combining complementary properties of the corresponding materials. We discuss further applications of these materials.

### Hybrid Hydrogels

Introduction of inorganic particles into hydrogel coatings allows the production of catalytically active interfaces (Agrawal et al., 2013), while on the other hand optical properties of hydrogels can be controlled through the addition of nanoparticles (Agrawal et al., 2013). Incorporation of magnetic nanoparticles into organic coatings has been used for the induction of release functionality (Hu et al., 2008) and manipulation of tissue for tissue engineering (Vidiasheva et al., 2018). Magnetic nanoparticles have also been used for adding magneto-responsive properties to magnetic hydrogels (Jaiswal et al., 2014). Stimuli responsiveness of hybrid interfaces produced by radical polymerization at the surface has also been shown (He et al., 2009).

**Figure 4:**
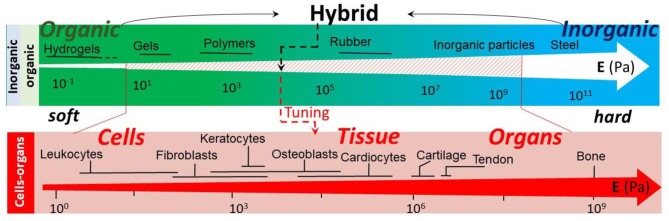
Mechanical properties (Youngs' modulus) of various constituents of organic-inorganic hybrid materials in relation to those of cells, tissue, and organs. Data are based on Kuznetsova et al. (2007) and Moeendarbary and Harris (2014).

Various functionalities and ways of incorporating inorganic contents (Zou and Kim, 2012) of organic/inorganic coatings have been shown, Figure 3. Properly designed inorganic/organic interfaces or hybrid (Schroeder et al., 2018) and functional hybrid (Sanchez, 2005) materials with special properties can address several biomedical challenges, including regeneration of bone tissues (Wang et al., 2012). The addition of nano- or macro- particles is beneficial for biomineralization, Figure 3. Calcium carbonate (vaterite) microparticles containing RGD peptide sequences can act as a template for stimulation of mineralization and mesenchymal stromal cell (MSC) differentiation *in vitro* and augment *in vivo* bone formation and impact on bone grafting (Green et al., 2009). Calcium carbonate has been applied in various areas. The crystallization process of calcium carbonate is complicated and includes the formation of different crystalline phases such as calcite, aragonite, and vaterite. Vaterite can hardly be found in nature and is an unstable polymorph (Shirsath et al., 2015). Porous vaterite calcium carbonate particles are spherical mesoporous polycrystals, with abundant advantageous properties like biocompatibility and high bio-macromolecule capacity, which is useful for drug delivery applications. Vaterite microparticles have also been utilized as a stabilizer in suspension polymerization in industrial settings and for regenerative medical approaches (Parakhonskiy et al., 2012; Shirsath et al., 2015). One of the most promising utilizations of these particles is as the active coating or efficient drug delivery, due to their entrance to micrometer-sized structures like cells and tissues (Parakhonskiy et al., 2012). Synthesis of CaCO_3_ particles with variable properties such as size, surface area, porosity, and hydrophobicity makes them a good candidate for surface coatings (Shirsath et al., 2015; Feoktistova et al., 2016) while the loading of bioactive macromolecules makes them attractive carriers for drug protection and release (Vikulina et al., 2018). The morphology and crystal form of calcium carbonate has been transformed, relating to protein-mediated nucleation during biomineralization (Xue et al., 2011). CaCO_3_-lentinan microspheres with a hierarchical composite pore structure have been produced by the self-assembly of nanoparticles. These structures could clearly decrease the release rate and prolong the release time of anticancer drugs, reducing potential side effects (Ma et al., 2013a). Hybrid crystals of CaCO_3_ with bovine serum (CaCO_3_/BSA) in the shape of a flying plate have been synthesized using nanoparticles. It has been illustrated that the nucleation and aggregation of the crystals affect the secondary structure of proteins, providing a promising way for encapsulation and delivery of different substances for pharmaceutical applications (Yang et al., 2009). It should be noted that biomineralization with calcium phosphate is also an important process (Cai and Tang, 2008). In addition, vaterite CaCO_3_ crystals can serve as sacrificial templates to assemble bio-functional structures for drug delivery, such as mesoporous carriers made of PEG and proteins (Behra et al., 2012; Schmidt et al., 2014; Balabushevich et al., 2015, 2016). In addition to mechanical properties, surface functionalization of the coatings has been identified as an important functionality (Azevedo et al., 2005). In this regard, functionalization of the coatings with enzymes and proteins has been identified to stimulate and promote cell growth. Recently, the addition of ALP (alkaline phosphates) on the surface of hybrid scaffolds has been shown to promote the cell adhesion (Muderrisoglu et al., 2018), where functionalization of titanium implants modified with hydrogels and calcium carbonate particles resulted in ~1.4 times higher cell viability. Antibacterial properties of the coatings have always been an important attribute of the coatings. Enhancing them by adding green materials, for example, pectins is seen as a significant development (Douglas et al., 2019). It can be stated that hybrid organic-inorganic coatings are continuing (Rezwan et al., 2006) to attract significant attention, particularly in tissue engineering, and mechanical properties is an important criterion here. Traditionally, tuning the mechanical properties plays a prominent role in controlling the cell adhesion. The addition of nanoparticles to a polymeric matrix has been linked to the formation of additional chemical bridges with polymers, resulting in enhanced mechanical properties (Bedard et al., 2009b) which, in turn, needs to be tuned for cell and tissue adhesion, Figure 4. Functionalization of polymeric films and coatings with remotely activatable microcapsules opens up further possibilities for drug delivery from the coatings (Volodkin et al., 2009a). Metal nanoparticles can also serve as local heating centers (Skirtach et al., 2005), which are shown to guide cells on a polymeric/nanoparticle surface (Kolesnikova et al., 2012) and can also selectively control polymer surfaces using a laser (Skirtach et al., 2010) releasing molecules adsorbed on their surface (Volodkin et al., 2009b). Morphological surface modifications represent another desirable functionality; here gradient coatings (Pinchasik et al., 2014) as well as recently proposed sponge-like structures (Manda et al., 2018) are seen as important functional building blocks. Tuning mechanical properties is identified as a very important functionality enhanced by adding inorganic particles to the polymer matrix. This is tailored to match materials necessary to host various cells, which possess very different mechanical properties, Figure 4. It can be seen from Figure 4 that by adjusting the inorganic fraction (weight percent), organic coatings can be used to adjust the mechanical properties matching those of cells. Enhancement of mechanical properties by combining organic molecules with inorganic nanoparticles has been shown (Schmidt et al., 2012). A similar effect has also been observed by adding carbon nanotubes (Yashchenok et al., 2010) and by precipitating carbon nanotubes with calcium carbonate particles (Chojnacka-Gorka et al., 2016). Furthermore, very different filler, nanocellulose, has also been applied to enhance mechanical properties of soft coatings (Lee et al., 2014). Investigation of the influence of the inorganic fraction on mechanical properties of softer coatings has also been carried out (Fu et al., 2008).

### Hybrid LbL Materials

Enhancement of mechanical properties, additional sensor functions, catalysis, and remote release capabilities have been implemented through the incorporation of nanoparticles in polyelectrolyte multilayer capsules and films, even in early research on nanoparticles in LbL layers (Yu et al., 2003). Nanoparticles, which form additional bonds and are generally stronger, have been incorporated in the shell of microcapsules and a remarkable increase of the Young's modulus has been observed, by pressing on them with a colloidal probe AFM (Bedard et al., 2009b). Subsequently, the addition of carbon nanotubes have been used to both enhance the mechanical properties and control the permeability through capsules (Yashchenok et al., 2010). Sensory functions have been added upon the incorporation of quantum dots in the PEM network (Ionov et al., 2006), while catalytic properties of microcapsules have been implemented upon incorporation of silver nanoparticles in the shell of polyelectrolyte multilayer capsules (Skirtach et al., 2007). Remote release capabilities have been developed into a fairly extensive research area with numerous and continuously developing applications. They can be achieved by various stimuli (Skirtach et al., 2011), among which nanoplasmonics is performed by the laser action on nanoparticles incorporated into PEM layers of capsules. At first, silver (Skirtach et al., 2004) and gold (Radt et al., 2004; Angelatos et al., 2005; Skirtach et al., 2005) nanoparticles were tested, both enabling the release by increasing the localized temperature increase. Later on, the release was achieved using various laser wavelengths (Skirtach et al., 2008). Additionally, spatially- and directionally- selective release was realized on capsules (Bedard et al., 2009a). One of the first applications of release from microcapsules was of that inside living cells (Skirtach et al., 2006), which later on led to the investigation of the surface presentation of peptides relevant to immunology (Palankar et al., 2009). Subsequently, release was conducted in organisms, i.e., inside Hydra (Anbrosone et al., 2016) and *C. elegans* worms (Lengert et al., 2018). In addition to nanoplasmonics, magnetic nanoparticles have been used to induce release by a magnetic field. Subsequently, release has been realized by ultrasound, where metal nanoparticles in PEM layers increased the density of the shell. Furthermore, capabilities of organic/inorganic interfaces for LbL have been shown to act as sorbents of radionucleotides (Bratskaya et al., 2014), UV responsiveness of polymeric layers was shown to be enhanced by the addition of an inorganic content (Katagiri et al., 2009). The extension in dual responsiveness to UV and ultrasound by TiO_2_/polyelectrolyte layers has recently been demonstrated (Gao et al., 2016), while functionalization of phenolic networks with metals has allowed the increase of coating multifunctionality (Guo et al., 2014). Carbon based materials have also been applied to polyelectrolyte multilayer structures, where incorporation of graphene (Kulkarni et al., 2010) has added responsiveness to light (Potts et al., 2011; Kurapati and Raichur, 2013), while redox potential has been added by ferrocene (Wang et al., 2011). Inorganic quantum dots incorporated into polyelectrolyte multilayer capsules functioned as sensors (Nifontova et al., 2018, 2019). Bringing in inorganic content improved thermal properties of organoclays (Calderon et al., 2008) and mitigated the scaling of calcium carbonate (Sheikhi et al., 2018). A combination of polyelectrolyte polymers and brushes were reportedly enhanced by the addition of gold nanoparticles (Boyaciyan et al., 2018). Some other functionalities include the enhancement of thermal properties (Banjare et al., 2014) including that in LbL layers (Puhr et al., 2014). An essential need for modification by inorganic nanoparticles was fully felt on thick, so called exponential and gel-like coatings, often produced using PLL (poly-L-lysine) and hyaluronic acid (HA). The addition of nanoparticles (Skirtach et al., 2010) strengthened the otherwise weak, gel-like films and enabled delivery of biomolecules on cells, while adsorption of capsules to those films added drug delivery capabilities (Volodkin et al., 2011). Furthermore, PLL/HA films have been used for masking approximately half of an embedded capsule to produce Janus capsules(Delcea et al., 2011a). Here, nanoparticles absorbed on the surface of thick but soft PLL/HA films were used to tune the rigidity of the film, which allows to control the degree of protrusion of particles and to control the patchiness of produced Janus capsules (Kohler et al., 2012).

### Hybrid Block Copolymers and Polymersomes

In functionalization of polymersomes with inorganic agents the surface plays an important role (Egli et al., 2011). Responsiveness to light is a very desirable property of polymeric delivery vesicles, delivering and releasing sulforhodamine B upon exposure to ultraviolet light (Dinu et al., 2016). But responsiveness to light can also be used for propulsion and therapy, which is generated upon the asymmetric deposition of a thin layer of gold on an erythrocyte membrane modified polymersome shell (Shao et al., 2018). Modification of the polymersome shell with magnetic nanoparticles has been shown to control the release from such hybrid vesicles (Sanson et al., 2011). Another valuable additional property of magnetic nanoparticles added onto the surface of polymersomes, is the enhanced contrast agent function, for magnetic resonance (MR) imaging and drug delivery (Yang et al., 2018).

### Hybrid Polymer Brushes

Polymer brushes prepared by the end-grafting of chains to/from flat or curved surfaces can be organic or inorganic in nature. It was shown that small nanoparticles with good affinity to polymers interact with polymer brushes without aggregation, but if the interaction between the polymer brushes and nanoparticles is weak, then aggregation can take place (Kim and O'shaughnessy, 2002). Tenability of the properties of hybrid organic-inorganic brushes is one of the most desired and frequently used applications. Functionalization of polymeric brushes by nanoparticles takes place at the interface. There, nanoparticles can be either adsorbed onto the surface of brushes or they can be inserted into the brushes with such factors as pH, temperature, solvent, and the ionic strength affecting this process (Tokareva et al., 2006). The process of the swelling and shrinking of polymer brushes can be performed reversibly, in which case the nanoparticles will either be exposed or hidden into the interior of the brushes. Various nanoparticles have been added to brushes including metal Pt, Ag, Au, and semiconductor CdSe, with predominantly sensor-like functions (Ionov et al., 2006).

### Other Hybrid Materials

There are also other types of matrices not mentioned in the terminology above. One example is resins, which can either be of synthetic or plant nature and which can convert to organic compounds. Hybrid resin-based materials found applications not only in the automotive industry, but also as fillers in dentistry. Dentures are immersed into hybrid materials and then cured (Jafari et al., 2017). Some other interesting application examples of polymeric based hybrid materials are membrane and water treatment (Tripathi and Shahi, 2011), where metal and metal oxide nanoparticles have been used as the inorganic phase (Ng et al., 2013).

### Hybrid Lipid Membranes

Functionalization of lipid bilayers with inorganic nanoparticles has traditionally been important not only for a fundamental understanding of cell function, but also for its practical applications. Given the nanometer size of lipid bilayer membranes, typically only nano-sized objects have been used to functionalize the lipid membrane. Interactions between lipid bilayers and nanoparticles depend on (a) the nanoparticles: material and its oxidation state, size, shape, roughness, charge, hydrophobicity; (b) the stabilizers of nanoparticles used to retain colloidal stability; and (c) the interface between nanoparticle/stabilizers and lipid interface. All these interactions determine the dominant forces upon the interaction among van der Waals, electrostatic, steric, depletion, and solvent driven contributions. Some peculiarities of the interaction in a physiological medium are determined by the presence of the physiological buffer with ionic strength of 150 mM, implying that electrostatic interactions are screened at relevant distances. Since lipid bilayers are relevant for cells, the interaction of nanoparticles with living cells and organisms is of particular important. Here, the NCL (National Characterization Laboratory) has screened over 100 various nanoparticles and concluded that size, surface charge and hydrophobicity are the most relevant parameters with regard to biocompatibility (Mcneil, 2009). Functionalization of lipid membranes by nanoparticles provides a number of functionalities and remains of continuous interest (Chan and Král, 2018). Both the hydrophilic adsorption of nanoparticles on the other part of the membrane (Volodkin et al., 2009c) or the incorporation of nanoparticles into the hydrophobic core of lipid bilayers (Rasch et al., 2010) is possible. Increasing the stability of lipid membranes and liposomes has been one of the functionalities that is enabled by nanoparticles (Zhang and Granick, 2006; Michel et al., 2013), which is used in drug delivery. It is worth noting that the interaction between nanoparticles and liposomes can be controlled, for example, by halides (Liu et al., 2018). The lipid membranes were functionalized with silica nanoparticles serving the function of sensors and providing drug delivery (Zuccarello et al., 2016). Metal nanoparticles adsorbed on the surface of lipid membranes and liposomes have been widely used to control the permeability of lipid membranes. For example, an ionic current has been monitored upon laser illumination (Palankar et al., 2014; Urban et al., 2016) to gain a detailed understanding of the re-arrangements within lipid membranes, where the reversibility of the opening and closing of membranes upon turning a laser light on- and off-, has been demonstrated. Nanoplasmonics, or laser-nanoparticles interaction, has been used to study the phase transition of lipids (Urban et al., 2009) and has been reported to initiate the transport of molecules across the lipid membrane (Wu et al., 2008; Troutman et al., 2009; Volodkin et al., 2009c; Paasonen et al., 2010). Magnetic iron oxide nanoparticles on lipid membranes allowed to induce a triggered release from liposomes upon the application of a magnetic field (Amstad and Reimhult, 2012; Bixner et al., 2016a). Release from the so-called bilayer-decorated magneto-liposomes was realized by alternating current electromagnetic fields (Chen et al., 2010), while metal oxide nanoparticles have also been proposed for release (Wang and Liu, 2014). Another important class of lipid bilayer vesicles is exosomes, which are shed out by the cells. It is important to determine their composition, which can be linked to diagnostics of various diseases. Upon linking nanoparticles to the outer shell of exosomes, enhancement of a rather weak Raman signal was obtained by means of the surface enhanced Raman scattering (SERS), which allowed various types of exosomes to be distinguished (Stremersch et al., 2016).

### Hybrid Proteins and Enzymes, Carbohydrates, Nucleic Acids as Well as Bacteria and Cells

In hybrid materials, proteins and enzymes, carbohydrates and nucleic acids are often used in the organic-in-inorganic assemblies (Alvarez-Paino et al., 2013; Umemura, 2015; Elzoghby et al., 2016; Compostella et al., 2017; Vetro et al., 2017), this falls into the subject of modification of the surface of nanoparticles. But inorganic nanoparticles find a distinct application niche in inorganics-in-organics assemblies. To fully exploit diversified properties on inorganic nanoparticles adsorbed onto or incorporated inside above-mentioned materials, it is essential to control their spatial distribution or self-assembly. There are various approaches to achieve that, for example, by adding polymers to nanoparticles for controlling their distribution and self-assembly (Parakhonskiy et al., 2010). In this regard, nucleic acids, specifically DNA molecules, were shown to drive molecular self-assembly at the nanometer scale (Rogers et al., 2016). Furthermore, DNA molecules can be used for assembling gold nanoparticles, for example, chiral structures (Kuzyk et al., 2012). In addition, it was also shown that particles, including magnetic particles, can be used as sensors for in microrheology (Ziemann et al., 1994).

Modification of bacteria by nanoparticles is also very useful for sensing. Indeed, microbial identification and microbial interactions can be performed with a label-free Raman spectroscopy (Lorenz et al., 2017). In this area, a very refined investigation is dedicated to detect phenotypic heterogeneity of bacteria, where Raman spectroscopy offers advantages over flow cytometry (Heyse et al., 2019). Coating bacteria with inorganic noble metal nanoparticles facilitates the enhancement of the Raman scattering signal (Zhou et al., 2014).

Functionalization of cells has been performed with various inorganic nanoparticles. Magnetic nanoparticle functionalization of red blood cells (Brähler et al., 2006) was performed to enhance the efficiency of MRI detection. In such applications, red blood cells can be used to deliver medicine and nanoparticles (Delcea et al., 2012). Here, absorption of gold nanoparticles on the outer layer of red blood cells was reported (Delcea et al., 2012) bringing in remote release functionalities. In this case, red blood cells could be taken from a patient, loaded with a desired drug, modified with nanoparticles and injected back to the same patient with remote release enabled functionality. Thermolysis of leukemia cells was performed by laser-nanoparticle interaction (Lapotko et al., 2006). Nanoparticles were brought in proximity to cells for gene delivery (Arita et al., 2011). The outer membranes of mammalian cells have been also functionalized with gold nanoparticles and laser light has been can been used in this case to deliver specifically deliver molecules from the surrounding cell culture medium into desired cells with a pre-determined patterning only (Xiong et al., 2014). In the following step in this area, spatially selective transfection of the chosen living cells has been achieved (Xiong et al., 2017).

### Properties of Inorganic and Organic Constituents Making Them Perfect Complementary Materials

**Figure 5:**
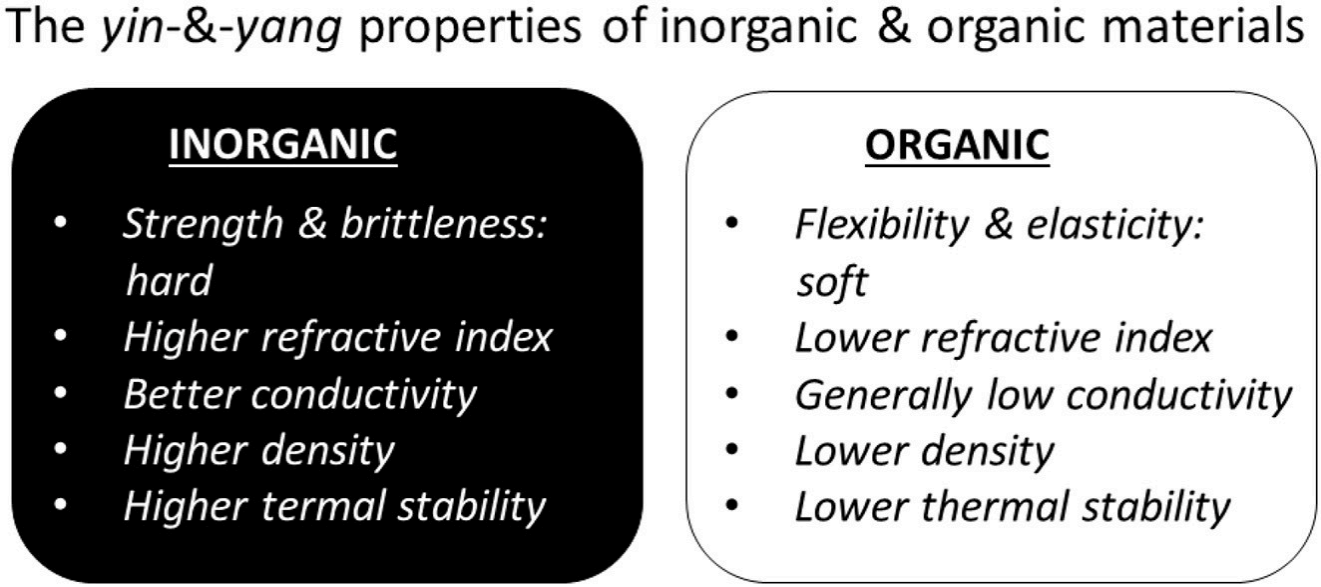
Antagonist (yin and yang), but complementary, properties of most common inorganic and organic compounds motivating their incorporation into hybrid materials.

By analyzing the above mentioned properties of hybrid materials, it should be noted that incorporation of inorganic constituents is made with a specific goal—to bring or complement missing properties, often these are mechanical strength, conductivity, optical/electrical/thermal properties or mass. A brief summary is outlined in Figure 5 to emphasize the contrast between these two groups of materials; this is similar to the organic vs. inorganic (oxide) materials discussed earlier (Sanchez et al., 2005). It can be noted that the same philosophy and the same properties drive the incorporation of organic materials into inorganic matrices (organics-*in*-inorganics), but the difference of approach is that research in the area of inorganics-*in*-organics is driven by a research community working with organic and soft matter, while research in organics-in-inorganics is put forward by researchers working and specializing mostly in inorganic materials and who use organic materials as additives. But, again, when designing hybrid materials, appropriate complementarity or treatment methods (Morent et al., 2009) are chosen and utilized to the advantage of both types of materials.

Overall, the choice of inorganic components would be determined depending on whether they possess these properties. Very often the added materials would add functionality associated with specific stimuli: physical, chemical, or biological (Delcea et al., 2011b).

A summary of some selected examples of hybrid inorganics-*in*-organics materials is presented in Table 1.

**Table 1:** Selected examples of hybrid inorganics-*in*-organics coatings presenting the composition, feature/functionalities and corresponding references.

**Organic content**		**Inorganic content**	**Features, functionalities, references**
Polymers	PLGA	Hydroxyapatite	Enhanced mechanical properties (Kang et al., 2011)
	Polyaspartate	CaCO_3_	Biomimetics (Sommerdijk and De With, 2008)
	PLL	Silica NP	Morphology control of biomimetics (Tomczak et al., 2005)
	PLA	Organoclays	Biodegradable bioplastics (Kasuga et al., 2001; Chang et al., 2003)
	PSS (polystyrene sulfonate)	TiO_2_	Catalysis, environmental applications (Priya et al., 2009)
	PCL	Hydroxyapatite	Stem cell growth (Priya et al., 2009)
	Cellulose	AgNP	Antibacterial properties (Perez-Masia et al., 2014)
	Silk fibroin	AuNP	Redox activity (Kharlampieva et al., 2009)
	Silk fibroin	Graphene	Enhancement of mechanical properties (Wang et al., 2016)
	Latex	Carbon nanotubes	Electroconductivity increase (Grossiord et al., 2008)
	PMMA, PVA, PLA, PAN, PBO, PA6, PDMS, epoxy	Carbon nanotubes	Reinforcement and theory of fiber reinforced composites (Coleman et al., 2006)
	PDMS-elastomer	Magnetic iron powder	Tuning surface roughness, wettability (Glavan et al., 2018)
	Styrene-butyl acrylate	Carbon black	Vibrational damping and electrical conductivity (Hu and Chung, 2011)
	MDMO-PPV	ZnO	Solar energy (Beek et al., 2004)
	Various polymers	Metal/metal oxide	Membrane and filtration (Tripathi and Shahi, 2011; Ng et al., 2013)
Hydrogels	Silk based injectable hydrogels	Hydroxyapatite	Enhancement of mechanics (Young's modulus 21 kPa), osteo-differentiation (Ding et al., 2017)
	Elastomeric (pHEMA) hydrogels	Hydroxyapatite	Stem cell differentiation (Song et al., 2009)
	Various hydrogels	Hydroxyapatite	Biomineralization (Cai and Tang, 2008)
	Gellan gum	CaCO_3_	Biomineralization (Douglas et al., 2016)
	Gellan gum	Montmorrilo-nite	Composition control (Lvov et al., 1996)
LbL polymers	PEI/PDADMAC/PAA	AuNP	Optical properties (Malikova et al., 2002)
	PSS/PAH	AgNP	Remote laser activation and release (Skirtach et al., 2004); catalysis and ultrasound (Skirtach et al., 2007)
	PSS/PAH, PSS/PDADMAC	AuNP	Remote laser activation (Radt et al., 2004; Angelatos et al., 2005) and measurement of temperature rise (Skirtach et al., 2005)
	PSS/PAH PDADMAC/montmorrilonite	Quantum dots	Sensors (Kharlampieva et al., 2010)
		AgNP	Mechanical and antibacterial properties (Cheng et al., 2017)
	PMAA (poly(methacrylic acid)	AuNR	Sensors (pH) (Kozlovskaya et al., 2008)
	PLL/HA	AuNP	Sensitivity to laser and enhanced mechanical properties (Volodkin et al., 2009a; Skirtach et al., 2010)
	PSS/PAH	Graphene oxide	Enhancement of mechanical properties (Kulkarni et al., 2010)
	IL-NH2	Graphene	Electro-catalysis (Zhu et al., 2010)
	PSS/PAH	Halloysite	Novel functionalization (Konnova et al., 2013)
	PUF/PEI/PAA	Halloysite	Flame retardant (Smith et al., 2018)
	PMMA/PS	Halloysite	Wear resistance (Song et al., 2016)
	PEI/PAA	TiO_2_	Dye- solar cells (Chen et al., 2013)
	PSS/NTA (nickel-nitrillotriacetic acid)	TiO_2_	Desorption of proteins (Andreeva et al., 2016)
	PSS with PEI as support	TiO_2_	Hydrophilic to hydrophobic conversion (Lu and Hu, 2016)
	PLA	Montmorrilo-nite	Mechanical properties (Svagan et al., 2012)
	PSS/PAH	CNT	Mechanical properties and release (Yashchenok et al., 2010)
	PSS/PAH and alginate	AuNP	Hydra, metazoan (Anbrosone et al., 2016); in *C elegans* (Lengert et al., 2018)
Polymer brushes	P2-VP	AuNP	pH sensing (Tokareva et al., 2006)
	Brushes	PtNP	Sensors (Mei et al., 2005)
	Brushes	AgNP	Sensors (Lu et al., 2006)
	Brushes	AuNP	Sensors (Lu et al., 2006), pH sensitivity (Boyaciyan et al., 2018)
	P2-VP	CdSe	Sensors (Ionov et al., 2006)
Block co-polymers and polymer-somes	Poly(trimethylene carbonate)-*b*-poly(l-glutamic acid)	Magnetic nanoparticles	Magnetic resonance imaging and magneto-chemotherapy (Sanson et al., 2011)
	PNIPAM-based	Magnetic nanoparticles	Triggered release (Bixner et al., 2016b)
	PEG-PPO-PEG, PEG-PBD; PS-b-PAA	Magnetic nanoparticles	MR contrast agent (Yan et al., 2015) and delivery (Yang et al., 2018)
	Chitosan and heparin	Gold layer	Propulsion and therapy (Shao et al., 2018)
Lipids	Liposomes	AuNP	Multiple reports on permeability changes and release of contents (Wu et al., 2008; Troutman et al., 2009; Volodkin et al., 2009b; Paasonen et al., 2010)
	Lipid bilayer membranes	AuNR, AuNP	Ion current modulation of by laser-AuNP and AuNR (Palankar et al., 2014) & by AuNP (Urban et al., 2016)
	Liposomes (DOPC)	SiO_2_, ZnO, TiO_2_, Fe_3_O_4_	Light-controlled release (Wang and Liu, 2014)
	Lipid membranes, phospholipids, phosphate-dylcholine, liposomes	Magnetic NP	Targeted delivery and permeability control (Chen et al., 2010; Amstad et al., 2011)
	Membrane of red blood cells	AuNP	Remote laser activation and release (Delcea et al., 2012)
	Exosomes	AuNP	Diagnostics (Stremersch et al., 2016)
	Inside living cells	AuNP-polymeric capsules & AuNP	Release from AuNP-functionalized capsules: a) inside HeLa cells (Javier et al., 2006); b) in immunology (Palankar et al., 2009); c) from AuNP inside cells (Huschka et al., 2010)
	On membrane of living cells	AuNP	Delivery of biomolecules from outside inside neurons (Xiong et al., 2018) and cells (Xiong et al., 2017)
	On membrane of cancer cells	AuNP	Destruction of leukemia cells (Lapotko et al., 2006), HeLa cells (Javier et al., 2008), tumors (Lukianova-Hleb et al., 2014)
• Proteins • Polysaccharides • Nucleic acids	Alginate, pectin, carrageenan, xanthan	Montmorillo-nite, sepiolite, CNT	Enhancement of mechanical properties, sensors (Eduardo Ruiz-Hitzky (Editor) 2008)
	Pectin	Nanoclay	Environmentally friendly packaging (Vartiainen et al., 2011)
	Actin	Magnetic particles	Microrheology (Ziemann et al., 1994)
	Galactose	CNT	Pathogen binding (Xia et al., 2017)
	PVA	CoO, BiFeO_3_	Dielectric CoO (Das et al., 2018) as well as thermal & magnetic properties of BiFeO3 (Halder et al., 2018)
	Gelatin, collagen	Hydroxyapatite	Good cell response of stem cells (Raucci et al., 2018)
	Alginate	Sr	Tissue engineering (Catanzano et al., 2018)
	Gelatin, collagen, zein	Clay	Enhanced properties (Alcantara et al., 2012)
		Layered double hydroxides	Biocomposite non-viral vector (Desigaux et al., 2006)
	Chitosan	AuNP	Biosensors (Rocha-Santos, 2014)
		Montmorillo-nite	Enhancement stability (Wang et al., 2005)
		CaCO_3_	Biomimetics (Yao et al., 2011)
		Hydroxyapatite	Control of properties (Ren et al., 2018)
	DNA	AuNP	Sensors based on aggregation of NP (Storhoff et al., 1998)
	DNA	AuNP	Nanostoves for melting DNA (Stehr et al., 2008)
	Nucleic acids	Layered double hydroxides	Gene transfection (Kundu et al., 2017)
Red blood cells	Interior	Magnetic nanoparticles	Contrast for MRI (Brähler et al., 2006)
Red blood cells	Surface modification	AuNP	Release by laser light (Delcea et al., 2012)
Bacteria	Surface modification	AgNP, AuNP	Sensing and detection (Zhou et al., 2014)
Cells (living)	Surface functionalization	AuNP	Delivery of biomolecules from outside inside neurons (Xiong et al., 2018) and cells (Xiong et al., 2017)
Cancer cells	Surface functionalization	AuNP	Destruction of leukemia cells (Lapotko et al., 2006), HeLa cells (Javier et al., 2008), tumors (Lukianova-Hleb et al., 2014)
Other: dentures-polymers	Composites and polymers	Silicon dioxide	Filling in dentistry (Jafari et al., 2017)
Other: films	Hexadmethyldisiloxane	Quartz-like	Plasma-induced switching from organic to inorganic (Morent et al., 2009)

## Conclusions

Hybrid coatings incorporating both organic and inorganic materials maintain a prominent role in developing advanced applications, where the softness, flexibility, and functionality of soft matter matrix need to be complemented with hardness and responsiveness to external stimuli and other properties offered by inorganic components. In this review, we have described and analyzed:

- hierarchy and structural organization of the hybrid materials in general, identifying inorganics-in-organics (inorganic constituents modifying organic materials), the focus of this overview, and situating it in the overall hierarchical scheme;
- composition of inorganics-*in*-organics was also analyzed identifying and describing the following inorganic constituents: minerals, clays, metals, semiconductors, carbons, and ceramics modifying organic materials such as: polymers in general as well as hydrogels, layer-by-layer assemblies, polymer brushes, block copolymers, other materials (resins), lipids, proteins and enzymes, carbohydrates, nucleic acids as well as higher level organisms: cells, bacteria, microorganisms;
- a diverse range of applications of hybrid inorganics-in-organics was presented highlighting hybrid:
  1. chemically relevant molecules:
    - hydrogels where inorganic content has been used for biomineralization and enhancement of mechanical properties;
    - layer-by-layer assembly, in which inorganic nanoparticles have widely been used for the release of contents from capsules and coatings as well as for the enhancement of mechanical properties and sensor functions.
    - polymer brushes, where inorganic nanoparticles have been used as sensors and for the enhancement of mechanical properties;
    - block copolymers, where inorganic nanoparticles have been used for propulsion of polymersomes.
    - other materials, i.e., resins, where inorganic content has been used to cross-link the composite fillings in dentistry or to enhance resins in automotive and other industries; together with
biologically relevant molecules:
  - lipids, proteins/enzymes, carbohydrates, nucleic acids;
  - bacteria, cells and microorganisms.
- *yin* & *yang* antagonist properties (hardness <-> softness, brittleness <-> flexibility, conductivity <-> non-conductive nature of soft materials, high density <-> low density, high thermal stability <-> low thermal stability) determining complementarity of hybrid materials, Figure 5.

Research in the area of hybrid materials is prevalent in more than one research community: organic-in-inorganics (structures)—used in a research community mostly working with inorganic structures, organics-in-inorganics (colloids)—by scientists designing colloidal particles; inorganics-in-organics—by researchers working with polymers, soft matter, and bio- and chemical molecules. Providing the organizational framework for the overall area of hybrid materials is useful to share ideas, protocols and developments between these different research communities. Because what unifies them is the design of the best performing hybrid materials which are responsive to stimuli of choice (Delcea et al., 2011b). Critical mass of knowledge, diverse approaches of the above-mentioned research communities and the ideal combination of yin-and-yang properties of organic and inorganic materials, points to a bright future of research in the area of hybrid materials.

## Outlook

Generally, attractive opportunities are awaiting research in the area of hybrid materials, because of the extensive range of diverse properties of very complementary types of materials; the critical mass of researchers interested in the subject; the diversity of approaches of different research communities; the extensive multidisciplinarity of approaches used by researchers working in this area; the projected high demand from other research communities, for example, biological sciences, who tap into the potential of not only hybrid materials, but also the approaches used to work with them.

More specifically, further research is on-going in various fields of inorganics-*in*-organics to utilize the synergy between materials and research communities. In the area of hydrogels, development of biomineralization enrichment, where inorganic particles supply cross-linking ions, utilization of possibilities of remote modification (cross-linking) or laser activation would be beneficial. Hydrogels seem to be of paramount importance in a number of areas, particularly in tissue engineering, where control and adjustment of mechanical properties is a challenge. Modification of hydrogels by enzymes, proteins, active biomolecules as well as nanoparticles provide a rich environment for further enhancement of the intrinsic properties of the organic matrix and for the development of desirable properties. Antibacterial and anticorrosion functionalities, often achieved through the addition of active compounds in nanoparticles, are other important characteristics of material relevant in the biomedical and nanomedicine sector. In LbL, complementarity between organic and inorganic materials is expected to impact the design of advanced drug delivery vehicles and capsules, including remote release *in vivo*, control of reactions in micro-compartment volumes as well as further exploration of ways to produce LbL in a more simple and reliable fashion. The incorporation of nanoparticles is seen as an important mechanism to control mechanical properties and to enable the spontaneous and remote release of encapsulated biomolecules. LbL coatings on flat substrates also benefit from mechanical properties control, sensor functions, remote action of various stimuli obtained through incorporation of inorganic nanoparticles and nano-structures. Furthermore, development of gradient coatings will bring additional functionalities. In polymer brushes, the introduction of inorganic nanoparticles will further impact the control of micro- and macro- level properties, where sensor functions can be particularly remarked. In the area of block co-polymers and polymersomes, remote release and sensor functions are desired functionalities to be developed further using hybrids. In the case of polymersomes as well as other delivery vehicles, propulsion enabled by addition of inorganic nanoparticles will allow the development of advanced applications.

In the area of lipid bilayers, introduction of inorganic nanoparticles will help to better understand fundamental mechanisms of lipid membrane functioning, which will be useful not only for fundamental science, but is also expected to impact drug delivery. Liposomes, particularly with the development of the so-called “stealth” liposomes, are effective delivery vesicles, while their modification by inorganic nanoparticles would further extend the range of release capabilities. Such other biologically relevant molecules, for example DNA, can be utilized for self-assembly of inorganic nanoparticles, which can eventually be used to build advanced sensors. Label-free sensing is also relevant for bacteria and inorganic nanoparticles can provide necessary enhancement. In cell biology, either of the constructs described above can be used, or inorganic nanoparticles can release from cells, providing further effective ways of delivering drugs. In addition, analytical methods will allow the tailoring and control of the cell adhesion, where the properties of inorganic nanoparticles and nanostructures are difficult to replace.

A large number of upcoming developments, well-positioned and interdisciplinary in nature, will both contribute and benefit from a perfect synergy between organic and inorganic materials. In short, the outlook is bright for hybrids.

## Author Contributions

MSS, KE, and AA contributed to writing and performed some experiments, on which this work is based. TELD, DV, BVP and AGS have organized work and led some of research directions, on which this work is based.

### Conflict of Interest Statement

The authors declare that the research was conducted in the absence of any commercial or financial relationships that could be construed as a potential conflict of interest.
